# Personalised energy targeting during venovenous ECMO: serial assessment of resting energy expenditure reveals phase-dependent metabolic demand and systematic discordance with predictive equations

**DOI:** 10.1186/s13054-026-06334-w

**Published:** 2026-09-16

**Authors:** Sarah K. Wilke, Niklas Behnel, Steffen Weber-Carstens, Andreas Edel, Michael Müller, Arne Mahlau, Robert Falk, Martin Ruß, Tobias Wollersheim

**Affiliations:** Department of Anesthesiology and Operative Intensive Care Medicine (CCM/CVK), Charité – Universitätsmedizin Berlin, Freie Universität Berlin and Humboldt-Universität zu Berlin, Augustenburger Platz 1, 13353 Berlin, Germany

**Keywords:** Extracorporeal membrane oxygenation, Calorimetry, Indirect, Energy metabolism, Nutritional status, Critical care

## Abstract

**Background:**

In patients receiving extracorporeal membrane oxygenation (ECMO), metabolic demand varies dynamically across the disease course, while conventional indirect calorimetry in ECMO patients underestimates measured energy expenditure (measured EE) because a substantial proportion of gas exchange occurs across the membrane lung. Specialised protocols can overcome this limitation and measure EE directly during ECMO. However, no adult study has yet compared serial measured EE measurements with concurrent energy intake to assess feeding equipoise or to quantify the temporal variability that personalised nutrition must address.

**Methods:**

Post-hoc analysis of prospectively collected data from 9 adult patients receiving venovenous ECMO for acute respiratory distress syndrome at Charité — Universitätsmedizin Berlin. Measured-EE was assessed using the MEEP protocol (75 valid measurements). Feeding equipoise was assessed as the ratio of actual energy intake to measured EE (equipoise: 80–110%). Discordance analysis compared measurement-based classification with hypothetical ESPEN-guided nutrition (25 kcal/kg/day) using adjusted body weight (primary) and actual body weight (sensitivity analysis).

**Results:**

Median measured EE was 1923 kcal/day (interquartile range, 1594–2222). The ECMO circuit accounted for 60.5% ± 18.2% of total gas exchange. Feeding equipoise was achieved on only 30 of 75 measurement days (40%); patients were overfed on 44% and underfed on 16%. ESPEN 25 kcal/kg/day with adjusted body weight misclassified feeding equipoise on 55% of days (concordance 45%); actual body weight yielded concordance of 49%. The respiratory quotient (0.908 ± 0.131) did not correlate with the feeding ratio (ρ = 0.024).

**Conclusion:**

Both actual feeding practice and standard predictive equations failed to match measured energy requirements regardless of body weight choice. The substantial intra-individual measured EE variability and the progressive decline in extracorporeal gas exchange contribution support a phase-specific, personalised approach guided by serial assessment of measured EE rather than static weight-based targets.

## Introduction

Adequate nutritional therapy in critically ill patients remains a central challenge in intensive care medicine. Both underfeeding and overfeeding are independently associated with adverse outcomes, including increased infectious complications, prolonged mechanical ventilation, and mortality [1–4]. The importance of matching energy delivery to individual metabolic demands is widely recognised—yet predictive equations fail to achieve this reliably. In the largest validation study to date, Zusman et al. compared predictive equations against 3573 indirect calorimetry measurements in 1440 ICU patients and found that equations achieved agreement within ± 10% of measured values in only one third of cases [5]. 

International guidelines—most prominently those of the European Society for Clinical Nutrition and Metabolism (ESPEN)—therefore recommend indirect calorimetry (IC) as the reference standard for determining resting energy expenditure (REE) in ICU patients [6, 7]. ESPEN further specifies that for patients with body mass index (BMI) greater than 25 kg/m², adjusted body weight should be used when applying weight-based Eq. [6]. Yet weight-based targeting is itself uncertain in the critically ill: body weight is frequently estimated rather than measured in the ICU, and neither admission weight nor standard equations account for the shifts in body composition—loss of muscle mass, fluid accumulation—that occur over the course of critical illness. A recent meta-analysis demonstrated that IC-guided nutrition therapy is associated with lower mortality and morbidity in critically ill patients [8]. Despite this, IC remains underutilized [9]. Moreover, even when EE is measured accurately, it does not by itself define the energy that should be delivered: measured expenditure does not distinguish the proportion of demand met by endogenous substrate mobilisation—which is not suppressed by feeding and remains immeasurable—from exogenous requirements, and the extent to which delivered nutrients are utilised varies across the phases of critical illness [10]. Measured EE therefore represents the starting point for individualised targeting, not the target itself.

In patients receiving extracorporeal membrane oxygenation (ECMO), a substantial and variable proportion of gas exchange occurs across the membrane lung, rendering conventional IC systematically inaccurate. Dresen et al., in the most comprehensive review of nutrition therapy during ECMO, concluded that the optimal quantity of energy supply in ECMO patients remains unknown, and that IC was historically deemed contraindicated because the oxygenator invalidates the measurement [11]. To address this limitation, several techniques have been developed: De Waele et al. described connecting a calorimeter to the oxygenator exhalation port, the MEEP protocol calculates membrane lung gas exchange from paired blood gas analyses, Pelekhaty et al. described the EPER protocol using a CO₂ sensor at the oxygenator port, and Tatucu-Babet et al. demonstrated modified IC feasibility in venoarterial ECMO [12–15]. Dresen et al. recognised these approaches as the only physiologically sound IC methods available during ECMO [11]. 

These methods have generated valuable data on EE under ECMO, but a gap remains in translating this information into clinical practice. Studies employing these protocols have characterised measured EE but did not document actual energy intake. Conversely, feeding equipoise studies in ECMO patients – most notably by MacGowan et al. (*n* = 203), Ohbe et al. (*n* = 1769), Ferrie et al. (*n* = 86), and Ridley et al. (*n* = 107) – have documented energy delivery but could only compare it against weight-based estimates, not against measured expenditure [16–19]. MacGowan et al. noted this limitation explicitly, observing that feeding to measured rather than calculated requirements may help limit such errors [16]. To date, no adult study has combined measured EE and actual energy intake in the same patients.

We therefore conducted the first integrated feeding equipoise analysis in adult ECMO patients. This study aimed to (a) determine whether actual energy delivery matches calorimetrically measured EE during venovenous ECMO, and (b) characterise the temporal dynamics of energy expenditure across serial measurements, in order to evaluate the appropriateness of static, weight-based targets in this population.

## Methods

### Study design and setting

This investigation is a post-hoc analysis of prospectively collected data from the MEEP study (Measuring Energy Expenditure in ECMO Patients; ClinicalTrials.gov NCT01992237; registered 25 November 2013), a monocentric observational study conducted at the ARDS/ECMO center of Charité — Universitätsmedizin Berlin, Germany. The study was approved by the institutional ethics committee (EA1/293/13) and conducted in accordance with the Declaration of Helsinki. This study is reported in accordance with the STROBE (Strengthening the Reporting of Observational Studies in Epidemiology) guidelines. Written informed consent was obtained from patients or their legal representatives. Serial measurements were collected between January 2019 and March 2021. The parent study has previously yielded publications on the MEEP protocol itself, quantitative gas exchange monitoring, and the role of dissolved oxygen during venovenous ECMO [13, 20, 21]. 

### Study population

Eligible patients were adults (≥ 18 years) requiring mechanical ventilation as well as venovenous ECMO for severe acute respiratory distress syndrome (ARDS). From the original dataset of 82 measurements in 9 patients, 7 were excluded: 5 performed after ECMO explantation and 2 obtained while ECMO gas flow was 0 L/min. The final analysis comprised 75 measurements in 9 patients. A sensitivity analysis including all 82 measurements is reported in the Supplementary Material.

### The MEEP protocol

Energy expenditure was measured using the MEEP protocol as previously described [13]. Total oxygen consumption (VO₂) and carbon dioxide production (VCO₂) were calculated as the sum of native lung and membrane lung contributions. Native lung gas exchange was assessed via IC using a Cosmed Quark RMR indirect calorimeter (COSMED, Rome, Italy) connected to the ventilator circuit, with VO₂ and VCO₂ averaged over a stable recording period of at least 20 min. Membrane lung gas exchange was derived from simultaneous blood gas analyses at the ECMO inflow and outflow ports, with blood oxygen and carbon dioxide content calculated using the physiological dissociation model described by Dash et al.[22] Measured EE was calculated via the modified Weir equation. [23] All measurements were performed during haemodynamic and ventilatory steady-state conditions. As measurements were obtained during catecholamine support and ongoing nutrition and did not require a fasted or strictly normothermic state, the values reported here represent measured energy expenditure under the prevailing clinical conditions rather than energy expenditure under true resting conditions.

### Nutrition protocol

Nutritional therapy followed the pre-established ICU protocol and was not adapted to MEEP-derived measured EE. The energy target was 25 kcal/kg actual body weight per day, in line with institutional practice at the time. Continuous enteral nutrition was the preferred route and was advanced stepwise toward the full target after initial stabilisation. Gastric residual volume checked once per shift; in case of intolerance or high reflux, the rate was reduced and subsequently re-escalated, and prokinetic agents were used as required. When enteral nutrition could not be established or maintained, parenteral nutrition was added. Standard enteral formulations provided 1.25 kcal/mL or, for concentrated formulations, 2.0 kcal/mL; parenteral nutrition provided 1.06 kcal/mL. Non-nutritional energy sources—intravenous glucose and propofol (1.035 kcal/mL as a lipid emulsion)—were included in the calculation of total energy intake. Body weight was not re-measured at regular intervals during the ICU stay, and changes in body composition were not accounted for.

### Body weight for predictive equations

In accordance with ESPEN guideline recommendations [6], predictive energy targets were calculated using adjusted body weight for patients with BMI greater than 25 kg/m², defined as ideal body weight (IBW) + 0.25 × (actual body weight − IBW). For patients with BMI of 25 kg/m² or less, actual body weight was used. Six of 9 patients (67%) had BMI greater than 25 and thus received adjusted body weight calculations.

### Feeding equipoise and discordance analysis

Actual energy intake (enteral and parenteral combined) was recorded for each measurement day from clinical documentation. Feeding equipoise was assessed as the ratio of actual daily energy intake to measured EE. Measurement days were classified as equipoise (80–110%), underfed (< 80%), or overfed (> 110%), consistent with thresholds used by MacGowan et al. [16] We use the term feeding equipoise to denote agreement between delivered energy and measured energy expenditure (ratio 80–110%); this reflects numerical balance between delivery and expenditure and does not, by itself, establish metabolic adequacy. For the discordance analysis, each measurement day was classified under 3 reference standards: (a) measured EE, (b) ESPEN 20 kcal/kg/day, and (c) ESPEN 25 kcal/kg/day. Concordance was defined as agreement between the ESPEN-based and measurement-based classifications. Bland-Altman analysis was performed at the patient level using median measured EE per patient. The respiratory quotient (RQ = VCO₂(total) / VO₂(total)) and the fraction of total measured EE derived from extracorporeal gas exchange (ECMO fraction) were calculated for each measurement.

### Statistical analysis

Continuous variables are reported as median (interquartile range [IQR]) or mean ± SD. Correlations were assessed using Spearman rank coefficient. A 2-sided *P* < .05 was considered statistically significant. Given the exploratory nature of the study, no adjustment for multiple comparisons was applied. All analyses were performed using Python 3.11 (pandas, numpy, scipy, statsmodels, and matplotlib).

## Results

### Baseline characteristics

Nine patients (6 male, 3 female) were included, contributing 75 valid MEEP measurements (Table 1). Median age was 60 years (range, 36–69), median actual body weight was 80 kg (range, 60–98), and median BMI was 28 kg/m² (range, 21–31). Six patients (67%) had BMI greater than 25, resulting in adjusted body weights ranging from 61.1 to 82.6 kg. All 9 patients received venovenous ECMO for ARDS. Median ECMO duration was 16 days (range, 12–41). Six patients (67%) survived to hospital discharge or transfer.

**Table 1:** Baseline characteristics of included ECMO patients

Characteristic	Value (*n* = 9)
**Age**,** years — median (range)**	60 (36–69)
**Sex — male / female**,** n**	6 / 3
**Body weight**,** kg — median (range)**	80 (60–98)
**BMI**,** kg/m² — median (range)**	28 (21–31)
*Primary diagnosis*,* n*	
Viral pneumonia	5
Bacterial pneumonia	2
ARDS without pathogen detection	1
Pneumocystis pneumonia	1
**ECMO configuration**	VV-ECMO, *n* = 9
**ECMO duration**,** days — median (range)**	16 (12–41)
**ICU length of stay**,** days — median (range)**	28 (12–178)
**Outcome — survived / died**,** n**	6 / 3
**MEEP measurements per patient — median (range)**	7 (2–21)
**Total measurements analysed**,** n**	75

Data are presented as median (range) unless otherwise specified

Abbreviations: ARDS, acute respiratory distress syndrome; BMI, body mass index; ECMO, extracorporeal membrane oxygenation; ICU, intensive care unit; MEEP, Measuring Energy Expenditure in ECMO Patients; VV, venovenous

### Resting energy expenditure

**Fig. 1:**
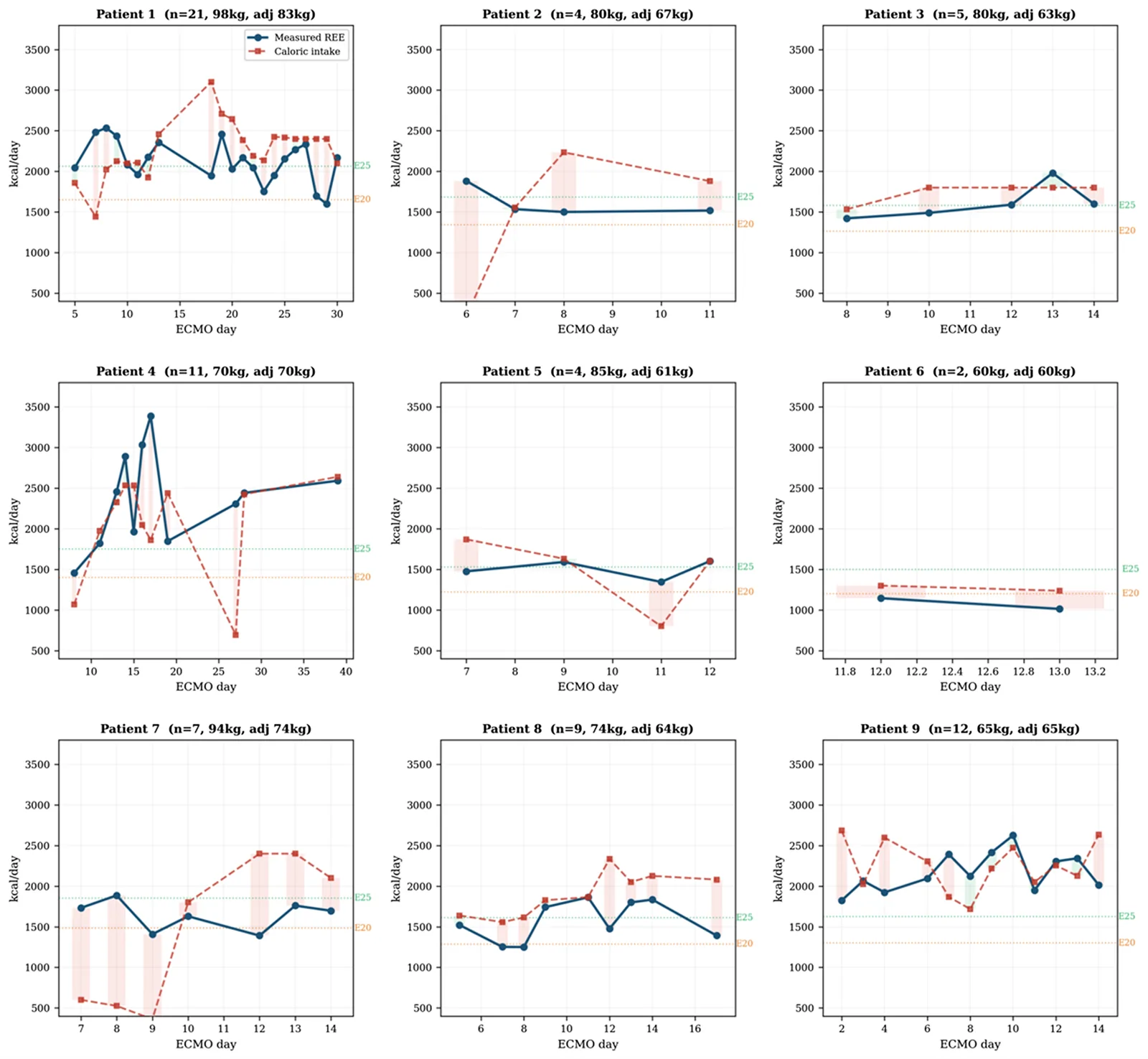
Serial trajectories of measured energy expenditure (EE) and actual energy intake per patient. Each panel represents 1 patient. Blue solid lines indicate measured EE (kcal/day); red dashed lines, actual energy intake. Horizontal dotted lines indicate ESPEN 20 (orange) and 25 (green) kcal/kg/day targets calculated using adjusted body weight for patients with body mass index greater than 25 and actual body weight otherwise. Actual energy intake comprises all delivered calories, including enteral and parenteral nutrition as well as non-nutritional sources (intravenous glucose and propofol)

Across all 75 measurements, median measured EE was 1923 kcal/day (IQR, 1594–2222; mean ± SD, 1936 ± 450; range, 1013–3389). Intra-individual variability was substantial, with the widest within-patient range spanning 1456 to 3389 kcal/day (a difference of 1933 kcal/day in a single patient; Table 2). The extracorporeal gas exchange accounted for a mean of 60.5% ± 18.2% of total measured EE (range, 24%–99.6%), declining over the course of therapy (Spearman ρ = −0.46; *P* < .001; Fig. 1).

**Table 2:** Measured energy expenditure and feeding equipoise per patient (*n* = 75 measurements)

Patient	*n*	Measured EE		Caloric	Feeding	Classification, *n* (%)			Cumulative
		Median	Range	Intake	Ratio (%)	< 80%	80–110%	> 110%	Balance
1	21	2,155	936	2,273	109 ± 24	2 (10)	10 (48)	9 (43)	+ 3,101
2	4	1,526	382	1,471	96 ± 60	1 (25)	1 (25)	2 (50)	−548
3	5	1,589	558	1,747	109 ± 11	0 (0)	2 (40)	3 (60)	+ 656
4	11	2,441	1,933	2,049	89 ± 31	4 (36)	5 (45)	2 (18)	−3,663
5	4	1,533	256	1,475	97 ± 28	1 (25)	2 (50)	1 (25)	−111
6	2	1,079	132	1,269	118 ± 6	0 (0)	0 (0)	2 (100)	+ 380
7	7	1,697	495	1,455	90 ± 60	3 (43)	0 (0)	4 (57)	−1,316
8	9	1,521	612	1,899	123 ± 20	0 (0)	3 (33)	6 (67)	+ 2,967
9	12	2,109	801	2,246	105 ± 22	1 (8)	7 (58)	4 (33)	+ 876
**All**	**75**	**1**,**923**	**—**	**—**	**104 ± 31**	**12 (16)**	**30 (40)**	**33 (44)**	**—**

Measured EE and energy intake values in kcal/day. Feeding ratio = actual intake / measured EE × 100. Classification: underfed (< 80%), equipoise (80–110%), overfed (> 110%). Cumulative balance = sum of (intake − measured EE) across all measurement days per patient. Abbreviations: measured EE, measured energy expenditure

### Feeding equipoise

The mean feeding ratio (intake/measured EE) was 104.3% ± 31.3%. Energy intake was delivered predominantly via the enteral route (70.9% of total intake across all measurement days), with parenteral nutrition contributing 24.8% and non-nutritional sources—intravenous glucose and propofol—accounting for 4.3%. At the individual level, the contribution of non-nutritional calories ranged from 0.0% to 12.1% across patients. Feeding equipoise (80–110%) was achieved on only 30 of 75 measurement days (40%). Patients were overfed (> 110%) on 33 days (44%) and underfed (< 80%) on 12 days (16%). At the individual level (Table 2), Patient 8 was consistently overfed (mean feeding ratio, 123%) while Patient 4 was predominantly underfed (mean ratio, 89%). The mean RQ was 0.908 ± 0.131 and did not correlate with the feeding ratio (ρ = 0.024; *P* = .84).

### Discordance analysis

**Fig. 2:**
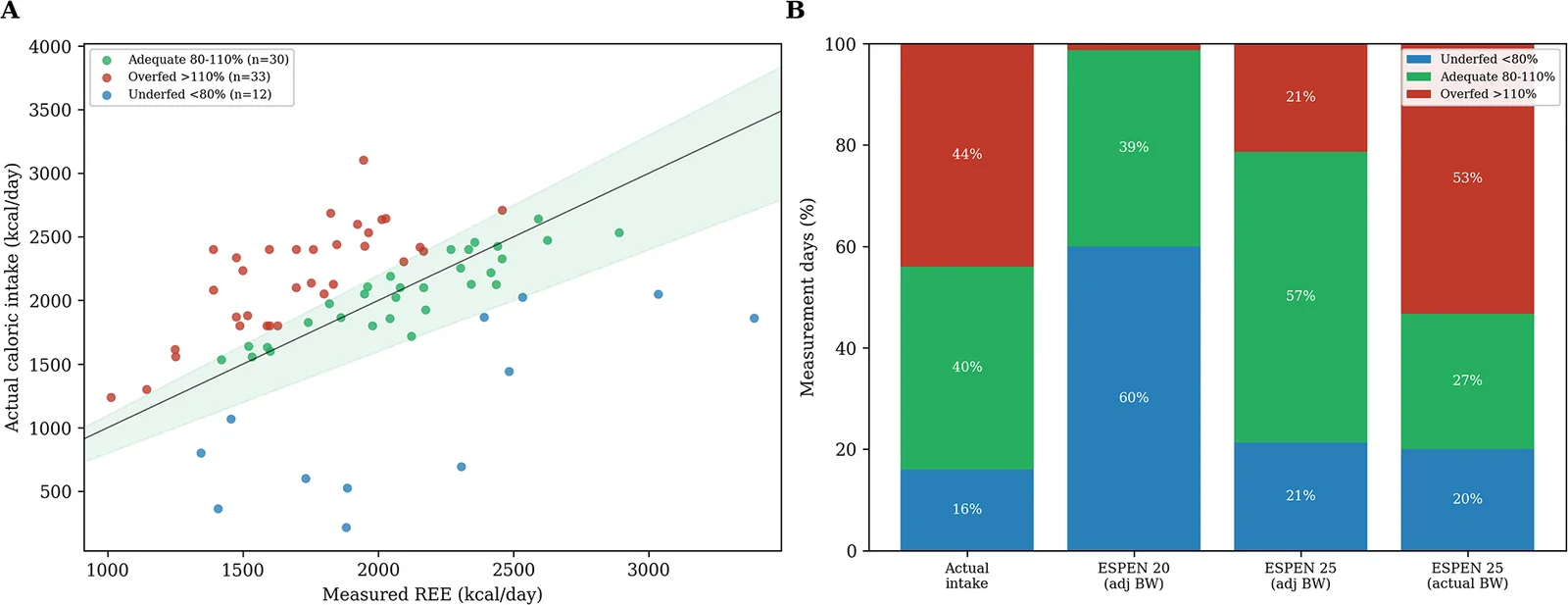
Feeding classification: actual versus ESPEN-guided. (**A**) Scatter plot of actual energy intake versus measured EE for each of 75 measurement days (green, equipoise 80–110%; red, overfed > 110%; blue, underfed < 80%). (**B**) Stacked bar chart showing feeding classifications under 4 scenarios: based on measured EE, ESPEN 20 with adjusted body weight, ESPEN 25 with adjusted body weight (primary analysis), and ESPEN 25 with actual body weight (sensitivity analysis). In panel B, the “Actual feeding” category denotes the measured actual energy intake delivered to patients, classified against measured EE; the remaining categories show classifications under the respective ESPEN-based predictive targets

In the primary analysis using adjusted body weight, Bland-Altman analysis revealed a mean bias of − 159 kcal/day for ESPEN 25 (limits of agreement, − 984 to + 665) and − 515 kcal/day for ESPEN 20 (limits of agreement, − 1335 to + 306). ESPEN 25 with adjusted body weight classified 21% of days as underfed, 57% as adequate, and 21% as overfed—yielding concordance with measurement-based classification of 45% (Fig. 2B). ESPEN 20 with adjusted body weight yielded concordance of 20%.

In the sensitivity analysis using actual body weight, ESPEN 25 yielded concordance of 49%, with a shift toward overfeeding (53% of days classified as overfed). Notably, although the clinical nutrition protocol itself targeted 25 kcal/kg using actual body weight, the distribution of actual intake differed from that of a strictly calculated ESPEN 25 (actual body weight) target: actual intake was adequate on 40% and overfed on 44% of days, whereas continuous application of calculated ESPEN 25 (actual body weight) would have classified 27% of days as adequate and 53% as overfed. This divergence indicates that uninterrupted delivery of the weight-based target would have resulted in lower, not higher, feeding equipoise. Concordance remained poor regardless of body weight choice (45% vs. 49%). A further sensitivity analysis including all 82 measurements (Supplementary Table S1) yielded essentially identical results (equipoise 41%, ESPEN 25 concordance 45%).

## Discussion

This study demonstrates, for the first time in an adult ECMO population, that neither actual clinical feeding practice nor standard predictive equations achieved consistent feeding equipoise when benchmarked against serial calorimetric measurements. Feeding equipoise was achieved on only 40% of measurement days. For context, feeding equipoise studies in non-ECMO ICU populations have documented similar challenges: McClave et al. reported that only 66% of ICU patients received at least 90% of their prescribed energy goal, and Reid found that 40–50% of ICU patient-days involved significant under- or overfeeding [24, 25]. In ECMO patients specifically, MacGowan et al. reported adequate energy delivery on approximately 63% of days in 203 venovenous ECMO patients – but assessed against estimated targets rather than measured EE [16]. The lower equipoise rate in our analysis, assessed against measured expenditure, suggests that a significant proportion of days classified as “adequate” under equation-based management may in fact represent over- or underfeeding relative to true metabolic demand.

### Discordance: the limits of static equations

The discordance analysis provides the most direct quantification of this problem. ESPEN 25 kcal/kg/day – recommended by both Dresen et al. and the 2025 ELSO Consensus Statement as the practical alternative when IC is unavailable—achieved correct classification on fewer than half of measurement days (concordance, 45% with adjusted body weight; 49% with actual body weight) [11, 26]. This concordance rate is comparable to the poor accuracy of predictive equations documented by Zusman et al. in 1440 general ICU patients [5]. The finding that concordance remained poor regardless of body weight choice has a specific implication: the problem does not lie in using the wrong body weight reference. Adjusting for BMI greater than 25 shifted the direction of the error - from predominantly overfeeding with actual body weight (53%) to a more balanced distribution with adjusted body weight (21% underfed, 21% overfed) – but did not improve the overall accuracy. This indicates that the discordance reflects the fundamental inability of any static equation to capture the dynamic metabolic variability observed under ECMO.

### Overfeeding despite interrupted delivery

A counterintuitive pattern deserves emphasis. Energy delivery in this cohort was repeatedly interrupted—by mobilisation out of bed, diagnostic transfers (computed tomography, angiography), surgical procedures, and episodes of enteral intolerance with high gastric reflux—which would be expected to produce systematic underfeeding. Yet actual intake exceeded measured EE on 44% of days, more often than it fell short (16%). Two factors reconcile this apparent paradox. Non-nutritional calories from intravenous glucose and propofol contributed modestly to total intake (4.3% across all measurement days) and did not account for the overfeeding pattern: their proportional contribution did not differ between overfed and equipoise days. Rather, the weight-based prescription itself, anchored to actual body weight, set a target that frequently exceeded measured expenditure; even partial delivery of an over-set target can surpass true demand. Consistent with this, a continuously applied calculated ESPEN 25 (actual body weight) target would have reduced rather than improved feeding equipoise (overfed on 53% of days). Overfeeding in this population is therefore not primarily a problem of excessive nutrition delivery, but of a target set too high relative to measured metabolic demand—precisely the error that serial measured EE assessment is positioned to correct.

### Phase-specific metabolic dynamics

Two observations from this dataset speak directly to the case for personalised, serial metabolic monitoring under ECMO.

First, intra-individual measured EE variability was substantial: patient-level ranges spanned from 1456 to 3389 kcal/day in a single patient (a within-patient difference of 1933 kcal/day; Table 2; Fig. 1), exceeding the magnitude of any plausible static correction factor. Such fluctuations in metabolic demand over the disease course – from the hypermetabolic phase of acute injury through stabilisation and early recovery – are well recognised in critically ill patients generally, and are not unique to those receiving ECMO. However, in the ECMO population these changes can only be detected through protocols that account for extracorporeal gas exchange; conventional IC at the ventilator alone would systematically underestimate true energy expenditure and obscure its trajectory. This temporal pattern is consistent with a conceptual framework of phase-specific nutrition therapy, which holds that energy and protein targets should be adapted to the patient’s metabolic phase rather than fixed at a single weight-based default [27]. The present data provide direct empirical support for this concept in the ECMO population.

**Fig. 3:**
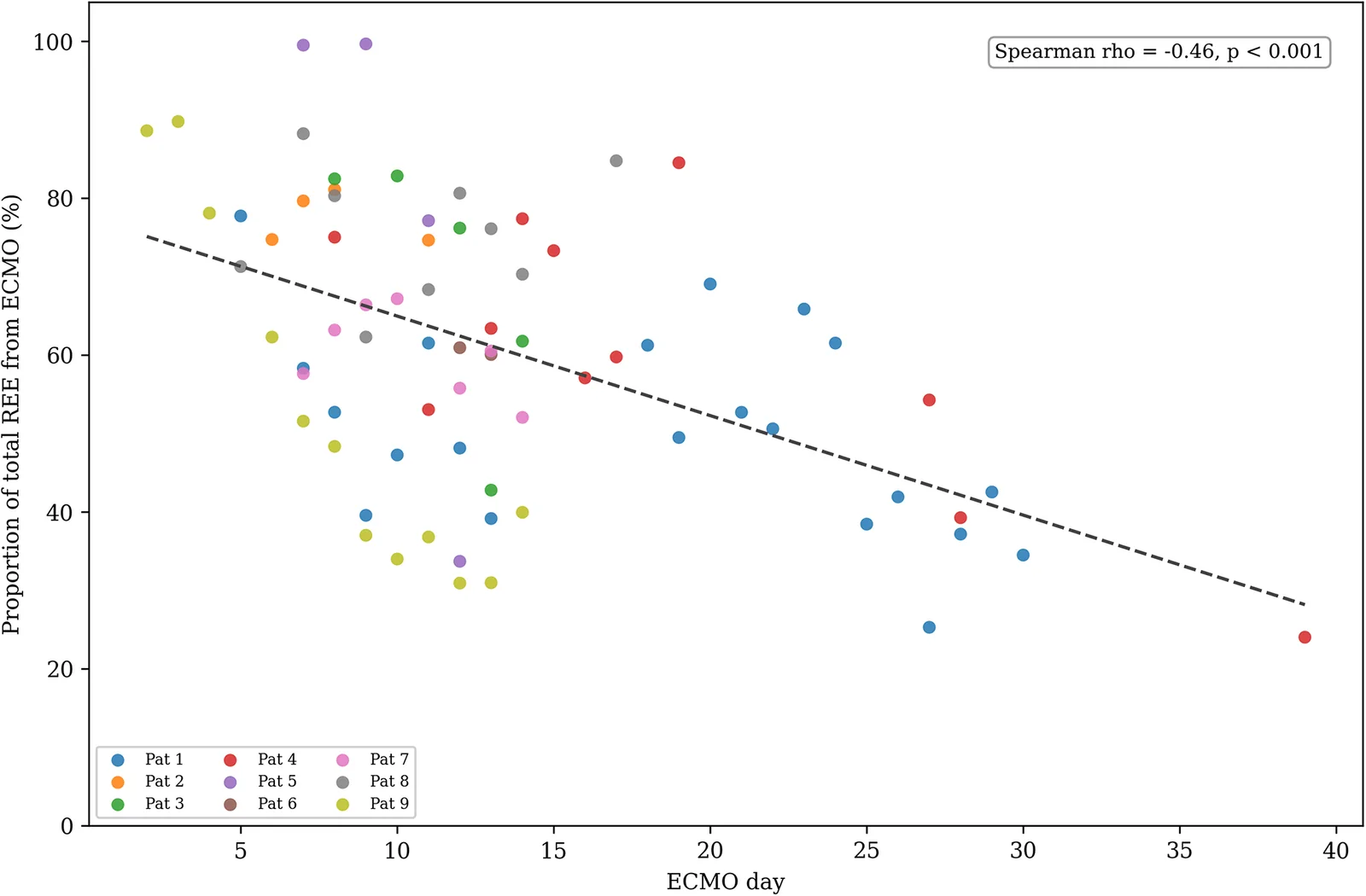
Proportion of total resting energy expenditure derived from extracorporeal gas exchange over the course of ECMO therapy. Each dot represents 1 measurement, colored by patient. Dashed line indicates linear regression. Spearman ρ = −0.46; P < .001

Second, the contribution of extracorporeal gas exchange to total measured EE declined significantly over the ECMO course (Spearman ρ = −0.46; *P* < .001; Fig. 3), reflecting progressive recovery of native lung function. This shift in the partition of gas exchange between the membrane lung and the native lung means that even the magnitude of error introduced by conventional indirect calorimetry – which captures only the native lung component – changes dynamically over time. A single measurement early in the ECMO course, when the membrane lung dominates, will underestimate EE far more than a measurement obtained during weaning. This adds a measurement-specific argument to the clinical argument above: not only does metabolic demand change, but the very tool used to approximate it changes in accuracy as well. Taken together, these findings indicate that serial assessment of measured EE is essential for individualised nutritional management in ECMO patients.

### ECMO contribution to gas exchange

The extracorporeal gas exchange accounted for a mean of 60.5% of total measured EE, confirming that conventional IC alone would have captured less than half of true metabolic demand. This proportion closely matches the 59.4% ± 24.6% reported by Pelekhaty et al. using the independently developed EPER protocol in 52 patients, providing cross-method validation [14]. 

### Respiratory quotient under ECMO

The absence of a correlation between RQ and the feeding ratio (ρ = 0.024) deserves comment. In non-ECMO patients, an RQ above 1.0 is typically interpreted as evidence of net lipogenesis from carbohydrate overfeeding. Under ECMO, RQ is inherently difficult to interpret, because sweep gas flow independently modulates CO₂ elimination across the membrane lung and partially decouples the measured RQ from whole-body substrate oxidation. Rather than indicating that RQ is an unreliable measurement, the lack of association is better read as a further sign of the limited metabolic meaning of the intake-to-measured-EE ratio: a ratio close to unity need not correspond to a physiologically appropriate substrate balance. Interpreted with due caution for these technical factors, RQ may still carry metabolic information, and its combination with other markers—rather than the feeding ratio alone—is more likely to reflect true metabolic handling.

### Methodological context

Our median measured EE of 1923 kcal/day is higher than the 1523 ± 432 kcal/day reported by Pelekhaty et al. [14] Several factors may contribute to this difference. The two studies employ different approaches to quantifying extracorporeal gas exchange: MEEP derives VO₂ and VCO₂ from blood gas analyses, whereas the EPER protocol measures gas concentrations at the oxygenator exhalation port. Although both approaches are based on fundamental physiological principles for determining extracorporeal gas exchange, neither has been validated under controlled ex vivo conditions to assess inherent methodological limitations. Consequently, discrepancies between results cannot be attributed to a single cause, as direct methodological comparisons between the approaches are lacking. Population differences may also play a role: our cohort consisted exclusively of venovenous ECMO patients with ARDS, whereas Pelekhaty et al. studied a mixed venoarterial/venovenous cohort. Despite these differences, both methods converge on the same key observation: predictive equations systematically fail to approximate measured EE. Pelekhaty et al. did not collect nutritional intake data; the present study complements their findings by providing the feeding equipoise dimension.

### Body weight and body composition

The persistence of poor concordance regardless of body weight reference points to a deeper limitation of weight-based targeting. Body weight in the ICU is frequently estimated rather than measured, and was not re-assessed at regular intervals in this cohort. More fundamentally, weight-based equations cannot account for changes in body composition that occur during critical illness—loss of skeletal muscle mass and accumulation of extravascular fluid—which alter the relationship between body weight and metabolically active tissue without being reflected in the weight used for calculation. ESPEN recommends adjusted body weight only for patients with BMI greater than 25 but provides no recommendation for tracking the compositional shifts that unfold over the disease course; bioelectrical impedance analysis is mentioned only as an option for assessing malnutrition risk [6]. In this context, a measured variable such as EE—however imperfect—offers a more direct reflection of current metabolic demand than any weight-derived estimate, reinforcing the rationale for serial calorimetric assessment alongside regular weight measurement.

### Measured expenditure versus nutritional target

A measured EE should not be equated with the energy that ought to be delivered. Indirect calorimetry quantifies total energy expenditure but does not distinguish the proportion met by endogenous substrate mobilisation—which is not suppressed by exogenous feeding and remains immeasurable at the bedside—from the proportion that must be supplied externally [10]. Particularly during the acute phase of critical illness, matching delivery to measured expenditure may therefore promote overfeeding, and the optimal target may lie below measured EE [28]. The individual energy target is further shaped by clinical parameters that calorimetry does not capture, including disease phase, glucose control and insulin requirement, serum phosphate, and catecholamine load. Whether delivered nutrients are actually utilised, and to what end, cannot be inferred from EE alone—an argument for complementing calorimetry with routine monitoring of glucose and phosphate rather than treating measured expenditure as a delivery prescription. These considerations do not diminish the value of serial EE assessment; rather, they define its proper role as the physiological starting point for an individualised, phase-adapted target rather than the target itself.

### Clinical implications

These findings have practical relevance for ECMO centers. The 2025 ELSO Consensus Statement recommended 25 kcal/kg/day as a pragmatic default [19], and our data confirm that this approach will misclassify more than half of patient-days. A recent review further identifies calorimetry-based, phase-adapted feeding as a priority in this setting and explicitly recognises adapted protocols such as the one used here as necessary for feasible serial monitoring of energy expenditure under ECMO, while noting that calorimetry-guided nutrition remains an open question for clinical outcomes [29]. The clinical significance of this misclassification is underscored by recent evidence that both underfeeding and overfeeding carry distinct risks in the critically ill, with overfeeding in particular associated with hepatic steatosis, increased CO₂ production, and prolonged ventilator dependence [27]. For centers with access to MEEP or comparable protocols, integration of serial measured EE assessments into nutritional management appears warranted. For centers without such access, our findings at minimum underscore the importance of close clinical monitoring and awareness that equation-based targets carry substantial uncertainty in this population. Whether calorimetry-guided nutritional adjustment improves clinical outcomes remains to be tested in future interventional studies.

These considerations point to a broader principle: no single parameter adequately captures metabolic state in these patients. Measured EE defines the magnitude of expenditure but not the appropriate delivery target, particularly given that endogenous glucose production can persist regardless of nutritional intake. RQ is difficult to interpret in isolation under ECMO, and the feeding ratio reflects numerical balance rather than metabolic appropriateness. This aligns with the concept, advanced in ESPEN monitoring recommendations, that indirect calorimetry is best understood as a metabolic monitor to be combined with clinical observation and a bundle of laboratory variables—including blood glucose and insulin requirement, serum phosphate, triglycerides, and urea—rather than used as an isolated target [30, 31]. Individually tailored nutritional therapy should therefore integrate serial measured EE with these complementary, phase-adapted markers, including potentially emerging catabolic indices such as the urea-to-creatinine ratio, rather than relying on any single measurement or fixed weight-based default [32]. Pending formal validation, particularly in ECMO patients, serial, trend-based interpretation of indirect calorimetry alongside these adjunct markers already represents a more defensible practice than fixed weight-based equations. Defining such a phase-adapted panel remains an important direction for future work.

### Limitations

To our knowledge, this dataset of 75 serial measured EE measurements with parallel nutritional intake documentation in adult venovenous ECMO patients is the first of its kind, and is best understood as proof-of-concept evidence motivating larger multicentre validation rather than a definitive epidemiological estimate. Several limitations apply. With 9 patients, this cohort—although, to our knowledge, the largest in which measured energy expenditure has been assessed serially under venovenous ECMO—does not permit definitive conclusions on any individual endpoint, and all findings should be regarded as hypothesis-generating. Patient-level generalisability and statistical power for between-patient comparison are inherently limited at this scale; the contribution of the dataset lies in the density of its within-patient serial sampling (75 measurements) rather than in the number of patients.

The study was designed as a longitudinal measurement series, but measurements could not be performed during every relevant period. Indirect calorimetry was not feasible during pulmonary deterioration when its technical preconditions were not met—for example, very low tidal volumes, an inspired oxygen fraction above 70%, or circuit leaks—nor during interventions or periods without a stable measurement state. Measurements were obtained only during haemodynamic and ventilatory steady state, defined as the absence of haemodynamic instability, a steady ventilatory pattern with low breath-to-breath variability, and no physiotherapy or positioning manoeuvres during the recording period. As a consequence, the most metabolically volatile periods are underrepresented, and the true intra-individual variability of measured EE is likely greater than observed. Individual measurements occasionally showed pronounced day-to-day variability in oxygen consumption and thus measured EE; where the underlying blood gas and calorimetric values were technically plausible, these measurements were retained rather than excluded, reflecting genuine variability in metabolic demand and the technical limits of single time-point assessment under ECMO. Feeding equipoise was assessed on a per-measurement-day basis, as measured EE and concurrent energy intake were available only on days when indirect calorimetry was performed. Days without a measurement were not captured, and the equipoise rate therefore reflects measurement days rather than the entire ICU stay; it should not be interpreted as the proportion of all patient-days on which energy targets were met. Measurement intervals were not standardised and were not obtained on consecutive days—the longest interruption was 7 days in a single patient—so the serial data represent discrete sampling points rather than continuous trajectories. Measurements began at a median of 7 days after ECMO initiation, leaving the earliest phase of support less well characterised.

Methodologically, the MEEP protocol relies on point blood gas measurements, which may not represent average gas exchange across the recording period and have been noted as a potential source of overestimation in blood gas–based methods. ^17^ Neither MEEP nor EPER has been validated against a gold standard under controlled conditions, and the contribution of factors such as dissolved oxygen to differences between approaches remains undetermined [21]. Body weight was predominantly estimated rather than measured and was not re-assessed at regular intervals, and body composition was not tracked. Measurements were not systematically assigned to the metabolic phases of critical illness (early acute, late acute, and post-acute), as the timing of each measurement was recorded relative to ECMO initiation rather than to the onset of critical illness; phase-specific energy requirements and the contributions of factors such as glucose and insulin levels, serum phosphate, renal replacement therapy, and catecholamine load were therefore not formally accounted for in the interpretation of measured EE. Finally, the study was not designed to determine whether calorimetry-guided nutritional adjustment improves clinical outcomes, and the single-centre design may limit transferability.

## Conclusions

In this longitudinal analysis of 75 serial calorimetric measurements in 9 venovenous ECMO patients with ARDS, feeding equipoise was achieved on only 40% of measurement days. ESPEN predictive equations - whether applied with guideline-compliant adjusted or actual body weight - misclassified feeding equipoise on more than half of days. The discordance persisted regardless of body weight reference, indicating that the problem lies in the nature of static equations applied to a population with dynamic metabolic demands. Not only does metabolic demand change across the disease course, but the shifting partition of gas exchange between native lung and membrane lung means that the accuracy of any single assessment method changes over time as well. These findings provide the first empirical link between variability in measured EE and feeding equipoise in adult ECMO patients and support a personalised, phase-specific approach to nutritional management guided by serial assessment of measured EE rather than static weight-based targets.

## Data Availability

The datasets generated and analyzed during the current study are available from the corresponding author upon reasonable request.
